# DEVELOPING AND TESTING A PROTOCOL FOR TELEPHONE REASSURANCE CALLS: A RANDOMIZED PILOT STUDY

**DOI:** 10.1093/geroni/igae098.0262

**Published:** 2024-12-31

**Authors:** Morgan Minyo, David Bass, Katherine Judge, Kate McCarthy, Christine Terry, Crysta Willis, Douglas Beach, Fatima Perkins

**Affiliations:** Benjamin Rose Institute on Aging, Cleveland, Ohio, United States; Benjamin Rose, Cleveland, Ohio, United States; Cleveland State University, Cleveland, Ohio, United States; Benjamin Rose, Cleveland, Ohio, United States; Western Reserve Area Agency on Aging, Cleveland, Ohio, United States; Western Reserve Area Agency on Aging, Cleveland, Ohio, United States; Western Reserve Area Agency on Aging, Cleveland, Ohio, United States; Western Reserve Area Agency on Aging, Cleveland, Ohio, United States

## Abstract

Telephone reassurance call programs that provide conversation and check-ins to isolated older adults have increased since the COVID-19 pandemic. The goals of these programs are to give older adults opportunities to express unmet needs, receive service referrals, and increase their use of home-and community-based services. Few empirical studies have examined the efficacy of these programs, including whether different approaches to telephone reassurance calls lead to different outcomes. This 24-month randomized controlled pilot study recruited older adults from an Area Agency on Aging to compared two approaches to telephone reassurance calls: 1) usual care, unstructured friendly check-in calls from a social worker (n=20) and 2) a newly developed protocol of semi-structured calls from a social worker (n=23), which was adapted from an evidence-based program. Multivariate results found significant differences in multiple outcomes. Compared to usual care (M=0.7, SD=1.0), participants who received the new semi-structured calls (M=2.1, SD=2.8) identified 1.75 more unmet service needs (t=2.69, p=.01). Participants who received the new semi-structured calls (M=1.1, SD=1.3) also received 1.23 more service referrals (t=4.07, p<.01) than usual care participants (M=0.3, SD=0.6). Additionally, participants who received the new semi-structured calls (M=3.7, SD=2.6) started or continued using 1.39 more services (t=2.02, p=.05) than usual care participants (M=2.4, SD=1.7). Findings suggest that the semi-structured protocol led to more beneficial outcomes while requiring only a seven-minute increase in the length of each telephone reassurance call. Discussion will focus on next steps for expanding the semi-structured protocol and sample in a multisite randomized trial.

